# Expression and function of the insulin receptor substrate proteins in cancer

**DOI:** 10.1186/1478-811X-7-14

**Published:** 2009-06-17

**Authors:** Katerina Mardilovich, Shannon L Pankratz, Leslie M Shaw

**Affiliations:** 1 Department of Cancer Biology, University of Massachusetts Medical School, Worcester, Massachusetts 01605, USA

## Abstract

The Insulin Receptor Substrate (IRS) proteins are cytoplasmic adaptor proteins that function as essential signaling intermediates downstream of activated cell surface receptors, many of which have been implicated in cancer. The IRS proteins do not contain any intrinsic kinase activity, but rather serve as scaffolds to organize signaling complexes and initiate intracellular signaling pathways. As common intermediates of multiple receptors that can influence tumor progression, the IRS proteins are positioned to play a pivotal role in regulating the response of tumor cells to many different microenvironmental stimuli. Limited studies on IRS expression in human tumors and studies on IRS function in human tumor cell lines and in mouse models have provided clues to the potential function of these adaptor proteins in human cancer. A general theme arises from these studies; IRS-1 and IRS-4 are most often associated with tumor growth and proliferation and IRS-2 is most often associated with tumor motility and invasion. In this review, we discuss the mechanisms by which IRS expression and function are regulated and how the IRS proteins contribute to tumor initiation and progression.

## Introduction

**Figure 1:**
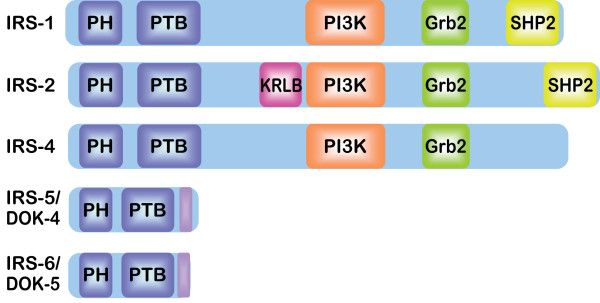
**Schematic of the IRS protein family**. Interaction domains of the IRS proteins are indicated. PH (purple), pleckstrin homology domain; PTB (purple), phosphotyrosine binding domain; KRLB (pink), kinase regulatory loop binding domain; PI3K (orange), region containing multiple PI3K binding motifs; Grb-2 (green), Grb-2 binding site; SHP-2 (yellow), SHP-2 binding site. IRS-5 and IRS-6 contain regions of similarity to each other in their C-termini (light purple).

The Insulin Receptor Substrate (IRS) proteins are a family of cytoplasmic adaptor proteins that were first identified for their role in insulin signaling. The first family member to be identified, IRS-1, was initially characterized as a 185 kD phosphoprotein that was detected in anti-phosphotyrosine immunoblots in response to insulin stimulation [1]. IRS-2 was discovered as an alternative insulin receptor substrate, initially named 4PS, in insulin-stimulated cells derived from *Irs-1*^-/- ^mice [2]. IRS-1 and IRS-2 are ubiquitously expressed and are the primary mediators of insulin-dependent mitogenesis and regulation of glucose metabolism in most cell types (reviewed in [3]). Humans express one additional family member, IRS-4, which is more restricted in its expression pattern and is found primarily in brain, kidney, thymus and liver [4]. A fourth IRS protein, Irs-3, is expressed in rodents, but not in humans [5,6]. More distantly related IRS family members IRS-5 and IRS-6, also known as DOK4 and DOK5, share homology in their N-termini, but have truncated C-termini [7-9] (Figure 1). Despite their significant homology, it is clear from the genotypes of knockout mice that the IRS proteins have non-redundant normal functions. *Irs-1*^-/- ^mice are born small and remain runted throughout their lives, implicating a role for this IRS protein in somatic growth regulation [10,11]. A similar contribution of the IRS homolog Chico to the regulation of cell size and growth in Drosophila has been observed [12]. Mice deficient for Irs-1 develop insulin resistance but do not progress to diabetes because they maintain normal pancreatic β-cell numbers. *Irs-2*^-/- ^mice are normal in size but have brain defects, the result of a 50% decrease in neuronal proliferation [13,14]. In contrast to *Irs-1*^-/- ^mice, Irs-2-deficient mice develop early-onset diabetes due to a combination of peripheral insulin resistance and a loss of β-cell function [13,15]. *Irs-2*^-/- ^females are also infertile, which together with evidence from insulin-signaling in *Drosophila *and *C. elegans*, supports a conserved mechanism for integrating reproduction and metabolism [16]. *Irs-4*^-/- ^mice are phenotypically normal, with only mild growth, reproductive and insulin sensitivity defects [17]. These differences in IRS function in normal development and physiology are also evident in cancer.

**Figure 2:**
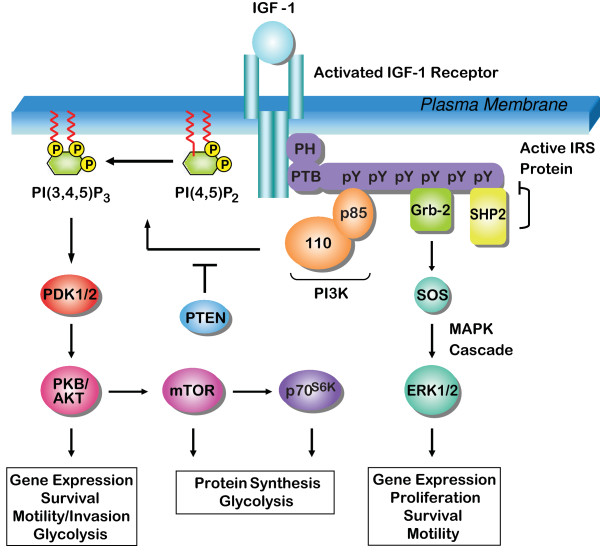
**Signaling via the IRS proteins**. The IRS proteins are recruited to activated cell surface receptors via PH/PTB domains in their N-termini. Once bound, they are phosphorylated on tyrosine residues in their C-termini. The phosphorylation of tyrosine residues (pY) creates docking sites for the recruitment of downstream signaling effectors. Subsequently, signaling cascades are activated that can regulate gene expression, protein synthesis, glycolysis, cell proliferation, survival and motility/invasion.

The IRS proteins contain no intrinsic enzymatic activity and they contribute to signaling through their function as adaptors to organize signaling complexes [18]. They share their highest level of homology in their N-termini, which contain two highly conserved domains that contribute to their recruitment to activated upstream receptors. The first of these domains is the pleckstrin homology (PH) domain. The PH domain is hypothesized to mediate both protein-protein interactions that facilitate the recruitment of the IRS proteins to receptors, and protein-phospholipid interactions that localize the IRS proteins to the cell membrane, in close proximity to transmembrane receptors [19-21]. The second conserved region is the phosphotyrosine binding (PTB) domain, which interacts with NPXY motifs in activated receptors [22,23]. An additional motif that contributes to receptor recruitment, the kinase regulatory loop binding (KRLB) domain, has been identified only in IRS-2 (Fig. 1)[22,24]. Upon binding to upstream receptors, the IRS proteins are phosphorylated on tyrosine residues in their C-termini, generating binding sites that recruit downstream effectors [25,26]. Effectors that have been characterized to bind to the IRS proteins include PI3K, Grb-2, SHP-2, Fyn, c-Crk, CrkII and Nck [9,27-32]. A recent study that utilized phosphorylated bait peptides to profile all potential phosphotyrosine dependent interaction sites in IRS-1 and IRS-2 identified additional potential interacting proteins [33]. However, additional studies will be necessary to determine if these effectors are recruited to the intact IRS proteins in response to physiological stimuli. It is the combined action of the downstream effectors that determine the signals that are transmitted through the IRS proteins and the cellular response that occurs (Figure 2). Importantly, many of these effector-signaling pathways have been implicated in tumorigenesis and cancer progression.

### Expression of the IRS proteins in human cancer

There are relatively few reports on the expression patterns of the IRS adaptor proteins in human tumors, either at the mRNA or protein level. The majority of these studies have evaluated the expression of IRS-1 and IRS-2, which are the more ubiquitously expressed family members in normal tissue. As an overall summary, IRS expression is most often elevated in human tumors when compared with normal tissue (Table 1). Expression of both IRS-1 and IRS-2 is reported to be increased in hepatocellular, pancreatic and prostate cancer and malignant pleural mesothelioma [34-40]. In other cancers, including breast, ovarian and medulloblastoma, only IRS-1 expression has been evaluated and a similar trend toward increased expression in primary tumors has been reported [41-44]. However, in breast cancer, which has been studied the most extensively of all cancers for IRS expression, there is also evidence that the expression of IRS-1 could correlate negatively with tumor progression. Specifically, IRS-1 is expressed at moderate to strong levels in normal tissue and well-differentiated carcinomas, but expression decreases in more poorly differentiated, higher-grade tumors [45,46]. Decreased IRS-1 expression is also observed in some non-small cell lung cancers (NSCLC), and this lower expression occurs more frequently in squamous cell carcinomas [47]. The conclusion drawn from the lung cancer study was that downregulation of IRS-1 may be an early event in NSCLC development. To date, the only study to examine IRS-4 expression in human cancer reported increased expression in hepatocellular carcinoma [39]. Finally, both IRS-2 and IRS-5 are upregulated at the level of gene expression in clear cell renal cell carcinoma [48]. One caveat to all of these expression studies, however, is that the IRS proteins can be phosphorylated on serine residues through negative feedback loops, which inhibits their function (reviewed in [49]). Therefore, expression of the IRS proteins may not reflect the functional status of these adaptor proteins. Additional studies are needed to establish clearly the expression and function of the IRS proteins in human cancer and to determine if their relative expression levels have prognostic or predictive value.

**Table 1:** IRS expression in human cancer

**Cancer Type**	**IRS** **Expression**	**Notes**	**Ref(s)**
Breast	↑ IRS-1	Well differentiated primary tumors	[41,43]
	↑ IRS-1	Nuclear IRS-1 correlates with good prognosis	[46]
	↓ IRS-1	Poorly differentiated, high grade tumors	[45,46]

Hepatocellular	↑ IRS-1	mRNA/protein increased in HCC	[38,39]
	↑ IRS-2	mRNA/protein increased in HCC	[38,39]
	↑ IRS-4	mRNA increased in HCC	[39]

Lung	↓ IRS-1	Larger (stage 1B) tumors, squamous cell carcinoma	[47]

Medulloblastoma	↑ IRS-1	Co-localized with polyoma JCV T-antigen in nucleus	[42]

Mesothelioma	↑ IRS-1	mRNA increased in malignant pleural mesothelioma	[37]
	↑ IRS-2	mRNA increased in malignant pleural mesothelioma	[37]

Ovarian	↑ IRS-1	Protein increased in malignant epithelial tumors	[44]

Pancreatic	↑ IRS-1	mRNA increased in 7/16 tumors	[34]
	↑ IRS-2	mRNA/protein increased in ductal-like cancer cells	[35]

Prostate	↑ IRS-1	Protein increased in tumors and metastases	[36]
	↑ IRS-2	Protein correlates with increased Myc expression	[40]

Renal	↑ IRS-2	mRNA increased in clear cell carcinomas	[48]
	↑ IRS-5	mRNA increased in clear cell carcinomas	[48]

### Upstream receptors implicated in cancer

The IRS proteins function as essential signaling intermediates downstream of many cell surface receptors that have been implicated in cancer. For example, the IRS proteins are major downstream effectors of the Insulin-Like Growth Factor-1 (IGF-1) receptor (IGF-1R) and they play a critical role in determining the cellular response to IGF-1 stimulation [50]. There is a strong correlation between enhanced IGF-1-mediated signaling and a wide range of cancers including malignancies of the breast, colon, prostate, thyroid, liver, pancreas and central nervous system [51]. In breast cancer, IGF-1 expression is elevated in the serum of patients and the IGF-1R is frequently over-expressed and is a prognostic indicator of tumor recurrence and reduced patient survival [52,53]. Other growth factor/hormone receptors that signal through the IRS proteins and that are associated with cancer include the insulin, prolactin, growth hormone (GH), and vascular endothelial growth factor (VEGF; KDR) receptors [54-56]. The IRS proteins have also been implicated in signaling downstream of the EGF receptor (EGFR), which may involve an EGFR/IGF-1R cross-talk [57,58]. Some integrin adhesion receptors also utilize the IRS proteins as signaling intermediates to relay intracellular signals [59-61]. In addition to surface receptors, several oncogenic fusion proteins that arise as the result of chromosomal translocations have also been reported to signal through the IRS adaptor proteins for their tumor promoting functions. These include the ETV6-NTRK3 gene fusion found in pediatric spindle cell sarcomas and secretory breast cancer, the RET-PTC3 gene fusion found in papillary thyroid cancer and the NPM-ALK gene fusion that is a transforming oncogene found in anaplastic large-cell lymphoma [62-64]. Full length anaplastic lymphoma kinase (ALK), a member of the insulin receptor superfamily and receptor for the growth factor pleiotrophin, also signals through the IRS proteins. ALK is expressed in breast and pancreatic carcinomas, melanoma and neuroblastoma, and has been demonstrated to be rate limiting for glioblastoma growth [65]. As common intermediates of many receptors that can influence tumor progression, the IRS proteins are positioned to play a key role in regulating the response of tumor cells to microenvironmental stimuli. As a result, they are also attractive candidates to be targets for interfering with the tumor-promoting signals that are initiated through these disparate receptors.

### IRS function in cancer

There are many studies on IRS function in human tumor cell lines and in mouse models that provide clues to the potential function of these adaptor proteins in human cancer. A general theme arises from these studies; IRS-1 and IRS-4 are most often associated with tumor growth and proliferation and IRS-2 is most often associated with tumor motility and invasion.

#### IRS-1

IRS-1 involvement in regulating tumor cell proliferation was foreshadowed by its role in somatic growth regulation. IRS-1 null mice are approximately 30% smaller than wildtype littermates and they maintain their runted phenotype throughout life [10,11]. IRS-1 is the predominant IRS family member that is activated by IGF-1 in well-differentiated estrogen receptor positive (ER+) human breast carcinoma cell lines [66,67]. IRS-1 mediates IGF-1-dependent growth in these cells, which has also been observed for hepatocellular and prostate carcinoma, medulloblastoma and malignant pleural mesothelioma cell lines [37,66-70]. The activation of both MAPK and PI3K signaling pathways has been implicated in the stimulation of proliferation by IRS-1 [67,69,71,72]. IRS-1-dependent signals also contribute to tumor cell survival. Suppression of IRS-1 expression by siRNA promotes apoptosis and renders ER+ breast carcinoma cells more sensitive to tamoxifen-stimulated cell death, whereas overexpression of IRS-1 confers resistance to TGF-β-induced cell death in hepatocellular carcinoma cells [73,74]. In contrast, Irs-1^-/- ^mammary tumor cells derived from mouse mammary tumor virus (MMTV)-polyoma virus middle T antigen (PyV-MT) mice are more resistant to apoptosis in response to serum deprivation than wildtype cells [75].

Transgenic mouse models have provided important information regarding IRS-1 function in cancer. Overexpression of IRS-1 in the mouse mammary gland results in mammary hyperplasia and tumorigenesis, which correlates with constitutive tyrosine phosphorylation of IRS-1, activation of Akt and Erk1/2 and association with β-catenin [76]. In mouse hepatocytes, overexpression of IRS-1 increases DNA synthesis and hepatic mass by 25%, further supporting the connection between IRS-1 and proliferation [77]. However, liver tumors do not develop in these transgenic mice, possibly because Fas-receptor is upregulated and this pro-apoptotic signal balances the pro-growth signals from IRS-1 [78]. The different results of these transgenic models with regard to tumorigenesis suggest that the oncogenic potential of IRS-1 may be dependent upon cellular context. Although IRS-1 overexpression promotes tumorigenesis, IRS-1 is not required for primary tumor growth as demonstrated by the fact that mammary tumor initiation and growth are not prevented or delayed in *Irs-1*^-/- ^mice in response to the PyV-MT antigen when compared with tumors that develop in wildtype littermates [79]. One important caveat to the IRS overexpression and knockout mouse mammary tumor studies is that the tumors that develop in both models are ER- and a possible preferential role for IRS-1 in ER+ tumor growth, which is suggested from the studies on human breast carcinoma cell lines, cannot be excluded. In contrast with the positive role for IRS-1 in early tumor development and growth, IRS-1 may play a suppressive role in tumor progression. Specifically, *PyV-MT*:*Irs-1*^-/- ^mammary tumors have a greater incidence and rate of lung metastasis when compared with *PyV-MT*:*WT *tumors [79,80]. Together with the IRS-1 expression data in human breast and lung cancer, these results reveal that loss of IRS-1 expression or function may facilitate tumor progression [45-47]. Once again, however, it is likely that IRS-1 function is cell context-dependent because deletion of Irs-1 in Apc^min-/+^(Min)/β-catenin-derived intestinal tumors decreases tumor incidence and growth and increases irradiation-induced apoptosis in the intestinal crypt [81].

#### IRS-2

The association of IRS-2 with tumor progression was first indicated by the finding that inhibition of the IGF-1R in ER- breast carcinoma cells, which express IRS-2 and lack or have decreased IRS-1 expression, does not inhibit tumor proliferation. However, inhibition of IGF-1R function does prevent metastasis of these cells in xenograft models [82,83]. Several studies have since demonstrated that IGF-1 promotes cell motility and invasion in human breast carcinoma cell lines and mouse mammary tumor cells that signal preferentially through IRS-2, but not in cell lines that express only IRS-1 [70,84-86]. A similar role for IRS-2-dependent signaling in cell motility and invasion has been reported for neuroblastoma and mesothelioma cells [37,87]. In contrast to IRS-2, IRS-1 may suppress cell migration because expression of IRS-1 in LnCAP prostate carcinoma cells decreases their motility. One possible mechanism by which IRS-2 contributes to tumor progression and cell invasion is by positively regulating aerobic glycolysis via the enhanced localization of the GLUT-1 glucose transporter on the tumor cell surface [88]. Similar to IRS-1, IRS-2 has also been implicated in promoting tumor cell survival, which is likely to contribute to its role in tumor progression. Irs-2-deficient PyV-MT-derived mammary tumor cells are significantly more sensitive to serum deprivation-induced apoptosis than wildtype tumor cells, and *Irs-2*^-/- ^tumors also have a higher *in situ *level of apoptosis [75]. Suppression of IRS-2 expression in hepatocellular carcinoma cells that express high levels of this adaptor protein also results in apoptosis [38]. Recent studies in pancreatic adenocarcinoma cells reveal that IRS-2 can regulate the expression of the IGF-1R to sustain high levels of IGF-1-dependent signaling [89]. Therefore, IRS-2 may promote tumor progression by stimulating a positive feedback loop to enhance IGF-1 signaling.

Additional support for IRS-2 as a positive regulator of tumor progression comes from *in vivo *mouse model studies. Mammary tumor metastasis is significantly diminished in *PyV-MT:Irs-2*^-/- ^mice, and Irs-1 deficient tumors that express elevated levels of active (i.e. tyrosine phosphorylated) Irs-2 have enhanced metastatic rates [75,79]. Irs-2 expression is elevated in tumors that arise in PTEN^+/- ^mice, and deletion of Irs-2 has no impact on tumor initiation, but it does suppress tumor growth and progression to invasive disease [40]. IRS-2, like IRS-1, can promote tumor initiation and progression when this adaptor protein is overexpressed in the mammary gland, a finding that would appear to conflict with the inability of IRS-2 to regulate tumor proliferation in human breast carcinoma cell lines [70,76]. One possible explanation for this discrepancy is that functions that are not observed at normal expression levels are gained when IRS expression levels are elevated. Alternatively, IRS-1 and IRS-2 may contribute to early tumorigenesis through distinct mechanisms. That is, IRS-1 may promote enhanced proliferation, whereas IRS-2 may promote enhanced survival, with the common outcome being tumorigenesis and progression.

The differential abilities of IRS-1 and IRS-2 to promote proliferation and motility/invasion, respectively, raises the question of how these similar proteins regulate divergent functions. IRS-1 and IRS-2 share approximately 35% identity in their C-termini where they recruit downstream effectors to phosphotyrosine binding motifs to initiate their signaling cascades. Many of the motifs are conserved between the two family members, and IRS-1 and IRS-2 have been reported to activate common signaling pathways including PI3K and the Erk1/2 MAPK kinases in a variety of cancer model systems [29,60,69,90,91]. Both of these signaling pathways have been implicated in promoting tumor cell proliferation, invasion and survival, but they cannot explain the differential abilities of IRS-1 and IRS-2 to regulate these functions. One potential mechanism for IRS-specific regulation of tumor cell functions is the recruitment of effectors to unique binding motifs in the C-termini of the IRS proteins that activate signaling pathways selectively downstream of either IRS-1 or IRS-2. For example, Rho-kinase (ROCK), which regulates cell adhesion and motility, is reported to be activated downstream of IRS-2 [91]. A recent proteomic analysis of potential IRS-1 and IRS-2 interacting partners provides further evidence that unique effectors can interact with each adaptor protein [33]. Distinct intracellular compartmentalization of IRS-1 and IRS-2 or differential sensitivities of IRS-1 and IRS-2 to negative feedback regulation would also impact the signaling outcomes of these adaptor proteins [49,92]. Additionally, acetylation positively regulates tyrosine phosphorylation of IRS-1 and negatively regulates tyrosine phosphoryaltion of IRS-2, which demonstrates that the function of these adaptor proteins can be differentially regulated by post-translational modifications [93-95].

An alternative mechanism for the differential involvement of IRS-1 and IRS-2 in cancer that would allow for the activation of a common signaling pathway by these adaptor proteins was revealed during the analysis of *Irs1*^-/- ^mammary tumors. Akt and mTor activation were enhanced significantly in *Irs1*^-/- ^mammary tumors when compared with the level of activation in wildtype tumors [79]. Although signaling through IRS-1 can activate these pathways in response to insulin or IGF-1 in many other model systems, when Irs-2 expression and function were compared between *Irs1*^-/- ^and *WT *tumors, Irs-2 activity was significantly higher in the absence of Irs-1, a finding that is mimicked by transient suppression of Irs-1 by siRNA *in vitro *[79]. A corresponding upregulation of Irs-1 expression and function is not observed in *Irs-2*^-/- ^tumors, or when Irs-2 expression is suppressed by siRNA (S. Pankratz, personal observation). Importantly, suppression of Irs-2 expression in *Irs1*^-/- ^tumor cells restores mTor activation to wildtype levels, confirming the contribution of Irs-2 to the increased mTor activity [79]. These *in vitro *findings support the hypothesis that Irs-2 compensates for the loss of Irs-1, and in doing so, enhances the activation of signaling pathways that promote tumor metastasis. A similar compensatory upregulation of Irs-2 expression in *Irs-1*^-/- ^mouse embryo fibroblasts has been hypothesized to explain the dominant role that IRS-2 plays in metabolic regulation [96].

#### IRS-4

IRS-4 expression increases in response to partial hepatectomy, a liver regeneration model, and expression is higher in hepatocellular tumors when compared with normal liver tissue [39,97]. In HepG2 hepatocellular carcinoma cells, suppression of IRS-4 expression by siRNA decreases IGF-1-dependent proliferation, which correlates with reduced Erk and p70S6-kinase activation [98]. Irs-4 has also been implicated in the insulin-dependent proliferation of a murine T-cell lymphoma cell line and it is overexpressed in a pediatric T-cell acute lymphoblastic leukaemia (T-ALL) that harbors a breakpoint fusion between the T-cell receptor beta locus and the IRS-4 gene [99,100]. These studies suggest that IRS-4 functions more similarly to IRS-1 than IRS-2 in cancer in that it is associated with tumor proliferation.

### Regulation of IRS expression

The evidence supporting the contribution of the IRS proteins to both tumor initiation and progression highlights the importance of understanding how the expression of these adaptor proteins is regulated. The differential expression patterns of the IRS proteins in both normal tissues and tumors support that their expression is likely regulated by unique mechanisms. Both the *IRS-1 *and *IRS-2 *genes are hormone-responsive, with *IRS-1 *regulated by the ER and *IRS-2 *regulated by the progesterone receptor (PR). Estrogen upregulates IRS-1 in ER+ breast carcinoma cells and IRS-1 expression decreases in response to the ER antagonists tamoxifen and ICI 182,780 [101-104]. This inhibition of IRS-1 expression may contribute to the suppression of breast cancer by these antiestrogens [101,102]. Progestin stimulation prior to IGF-1 treatment of PR+ breast carcinoma cells upregulates IRS-2 expression levels and tyrosine phosphorylation, thereby enhancing downstream IRS-2-dependent signals [105-107].

Non-hormone-dependent pathways also regulate the IRS genes. E-box elements in the *IRS-1 *promoter and proteins that bind to these elements positively regulate IRS-1 expression in HepG2 hepatocellular carcinoma cells [108]. E-boxes are often found in promoters of genes involved in metabolism and are consensus *cis*-elements for members of the basic helix-loop-helix family of transcription factors. IRS-2 is positively regulated by the cAMP-mediated activation of CREB, a pathway that is essential for the expression of this adaptor protein in pancreatic β-cells [109]. Members of the Forkhead transcription family, including FOXO1 and FOXO3a, can also positively regulate IRS-2 expression [110]. Several growth factor/hormone signaling pathways that are associated with cancer including fibroblast growth factor (FGF), epidermal growth factor (EGF) and insulin can modulate IRS-1 and IRS-2 expression levels [86,111-113]. The EGF-induced upregulation of IRS-2 expression occurs through a JNK/c-Jun/AP-1 pathway [86]. IRS-1 expression is negatively regulated by all-*trans *retinoic acid (ATRA), which arrests the growth of ovarian carcinoma cells in G_0_–G_1 _[44].

Amplified in breast cancer 1 (AIB1), also known as steroid receptor coactivator-3 (SRC-3), regulates both IRS-1 and IRS-2 expression [114,115]. AIB1 is an oncogene that is often overexpressed in human tumors and it promotes the growth of hormone-insensitive tumor cells through its action as a coactivator of nuclear receptors [116]. AIB1 directly regulates IRS-1 transcription by cooperating with the AP-1 transcription factor [115]. The importance of this IRS-1 regulatory pathway is demonstrated by the fact that deletion of AIB1 has a protective effect on mouse mammary glands against carcinogen-induced tumorigenesis, which can be explained in part by decreased IRS-1 expression and decreased Akt signaling [117]. The breast cancer-associated gene-1 (BRCA1) is a tumor suppressor that is mutated or deleted in 10% of hereditary breast cancers [118]. BRCA1 interacts directly with the IRS-1 promoter and inhibits transcription of the IRS-1 gene through epigenetic modification of histone H3 and H4 [119]. Deletion of BRCA1 in mice leads to increased expression of some members of the IGF-1 signaling pathway, including IRS-1 [119]. The association of IRS-1 expression with BRCA1 provides additional support for the involvement of this IRS family member in tumor initiation.

At the post-transcriptional level, two microRNAs, miR-126 and miR-145, have been identified that target and suppress IRS-1 protein expression [120-122]. Both miR-126 and miR-145 inhibit cell growth and their expression is frequently decreased in many cancer types [120,121,123,124]. Taken together, these findings are in keeping with a growth-promoting role for IRS-1 in tumors. miR-145 has also been implicated in positively regulating embryonic stem cell differentiation [125]. Interestingly, IRS-1 promotes stem cell self-renewal and its expression decreases during embryonic stem cell differentiation when miR-145 expression increases [126]. To date, miRNAs that target other IRS family members have not been identified.

### Feedback regulation of IRS function and expression

**Figure 3:**
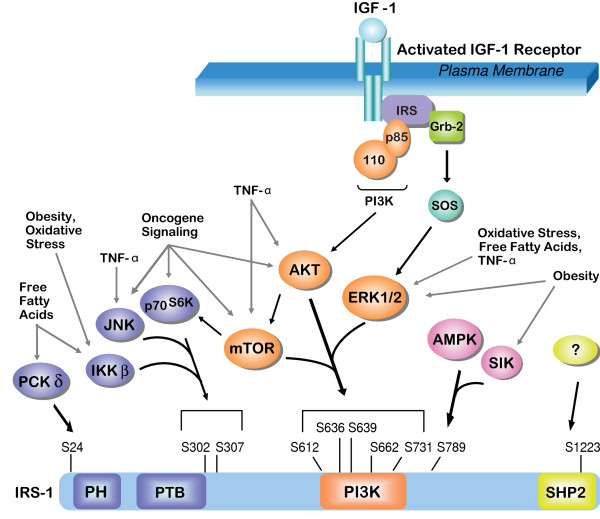
**Negative feedback regulation of IRS-1**. Serine residues that are phosphorylated in IRS-1 and the kinases that target these sites are indicated. Kinases shown in purple mediate signaling events that impede IRS-1 localization to the membrane or upstream receptors by disrupting PH and/or PTB domain function. Kinases shown in orange mediate signaling events that interfere with PI3K recruitment and activation. Kinases shown in pink mediate signaling events that result in phosphorylation of S789. Kinases that initiate signaling events that result in phosphorylation of S1223 and interfere with SHP-2 binding are unknown (yellow). Exogenous stimuli that have been implicated in cancer and that can activate kinases to regulate IRS-1 serine phosphorylation are indicated.

The expression and function of the IRS proteins can be regulated post-translationally. Negative feedback regulation of the IRS proteins by serine phosphorylation was first demonstrated in insulin-dependent signaling, and this feedback pathway is essential for regulating insulin sensitivity and glucose homeostasis by limiting the magnitude and duration of the insulin signaling response [49,127-129]. Serine phosphorylation of the IRS proteins interferes with their function by targeting these adaptor proteins for inactivation and/or proteasomal degradation (Figure 3) [49]. Phosphorylation on specific residues, such as serines 302 and 307, disrupts IRS-1 function by inhibiting interactions between the IRS-1 PTB domain and upstream receptors [130,131]. As a result, IRS-1 is not phosphorylated on tyrosine residues and cannot organize downstream signaling complexes [130,132]. IRS-1 and IRS-2 have also been shown to interact with 14-3-3 proteins through phosphoserine residues within the PTB domain [133]. Binding of 14-3-3 proteins to the PTB domain may physically prevent the IRS proteins from interacting with upstream receptors, which prevents IRS-mediated signaling.

Serine phosphorylation of the IRS proteins can also directly interfere with interactions with downstream effectors and selectively prevent their activation [131,134]. For example, phosphorylation of serine residues within the PI3K-binding region can inhibit interactions between the IRS proteins and p85, the regulatory subunit of PI3K [135]. In metastatic mouse mammary tumors, Irs-1 is phosphorylated on serine residues in the PI3K binding region and the association with p85 is decreased when compared with non-metastatic tumors [79]. Phosphorylation of serine-1223 in IRS-1 interferes with recruitment of the tyrosine phosphatase SHP-2 and, as a result, IRS-1 tyrosine phosphorylation is enhanced [136]. As mentioned previously, the distinct functions of IRS-1 and IRS-2 in tumor progression may reflect a differential sensitivity of IRS-1 and IRS-2 to the effects of negative feedback regulation, which could alter the longevity and intensity of signals initiated through each adaptor protein [112,137,138].

In addition to disrupting protein-protein interactions, serine phosphorylation of IRS-1 and IRS-2 can target these adaptor proteins for ubiquitination and degradation via the 26S proteasome [139-141]. This downregulation is mediated by an mTOR-dependent negative feedback loop that also involves p70S6-kinase [134,142-145]. In an extreme example of this negative feedback, tumors with constitutive activation of mTOR, such as those with mutations in the *TSC-1 *or *TSC-2 *genes, are benign and rarely progress to a more malignant state because both IRS-1 and IRS-2 are degraded and cannot sufficiently activate survival signals [143,144,146]. mTOR can also regulate proteasome-dependent degradation of the IRS proteins by stimulating the endoplasmic reticulum stress (ER-stress) response. Loss of TSC function and subsequent mTORC1 activation lead to ER-stress and activation of the unfolded protein response (UPR) [147]. Inhibition of ER-stress in TSC-1^-/- ^or TSC-2^-/- ^cells that have decreased expression of IRS-1 and IRS-2 results in increased IRS protein stability and insulin-induced tyrosine-phosphorylation, which leads to enhanced Akt activation. Likewise, induction of ER-stress dramatically increases IRS-1 ubiquitination-dependent proteasomal degradation [147].

A significant amount of research has focused on understanding the contribution of IRS serine phosphorylation to insulin resistance and diabetes and the information gained from these studies can be applied to cancer (reviewed in [49]) (Figure 3). For example, the inflammatory cytokine tumor necrosis factor-α (TNF-α) inactivates IRS-1 through a JNK-mediated phosphorylation of S307 (S312 in human IRS-1), which results in insulin resistance [127,131]. Inflammatory cells in the tumor microenvironment secrete TNF-α and other cytokines that positively contribute to tumor progression. Therefore, serine phosphorylation of the IRS proteins may be a mechanism by which the stromal microenvironment influences tumor behavior [148]. Additional exogenous factors that can regulate IRS serine phosphorylation levels and that are associated with cancer progression include elevated free fatty acids, obesity and oxidative stress [149-152]. Potential intrinsic mechanisms to phosphorylate and regulate IRS function include many kinases that are activated by oncogenic signaling, including mTor, Akt, multiple PKC family members, Erk1/2, S6-kinase, IKKβ, AMPK and SIK, as well as the aforementioned JNK [49,153]. Additional studies are warranted to determine the potential of these IRS feedback pathways as therapeutic targets for cancer treatment.

IRS signaling functions can be influenced by additional post-translational modifications including O-linked glycosylation and, as mentioned previously, acetylation. Increased activation of the hexosamine pathway (HBP) can induce O-glycosylation of IRS-1 and IRS-2, which decreases IRS tyrosine-phosphorylation and prevents activation of the PI3K signaling pathway [154,155]. Acetylation of IRS-1 and IRS-2 decreases or increases, respectively, their level of tyrosine phosphorylation and downstream signaling. IRS-1 deacetylation is mediated by HDAC2 and IRS-2 deacetylation is mediated by SirT1 [93-95]. However, neither of these IRS posttranslational modifications have been investigated in the context of cancer and it is not known if they contribute to the regulation of IRS-dependent signaling in tumor cells.

### Involvement of the IRS proteins in transformation

The transforming potential of the IRS proteins has been demonstrated in several different model systems, with most of the evidence coming from studies on IRS-1. The earliest indication that IRS-1 had oncogenic potential came from studies on *IGF-1R *null 3T3 fibroblasts (R- cells), which are resistant to transformation by a number of oncogenes, including SV40 T-antigen [156,157]. Overexpression of IRS-1 in these R- cells cooperates with both SV40 T-antigen and Src to promote transformation, whereas in wildtype 3T3 cells, suppression of IRS-1 expression inhibits SV40 T-antigen-mediated transformation [158,159]. Subsequent studies have demonstrated that overexpression of IRS-1 in 3T3 fibroblasts, independent of SV40 T-antigen, promotes growth in soft agar and tumorigenicity in nude mice [160]. IRS-1 also cooperates with V-HA-Ras to transform 32D murine hematopoietic cells [161]. IRS-1 tyrosine phosphorylation and activation of MAPK, SHP-2 and PI3K signaling pathways have been implicated in the mechanism by which this adaptor protein promotes transformation [161-163]. Overexpression of both IRS-1 and IRS-2 in immortalized mammary epithelial cells disrupts normal luminal differentiation and polarization and promotes dysregulated growth [76]. Moreover, as mentioned previously, transgenic overexpression of IRS-1 or IRS-2 in the mammary gland results in hyperplasia, tumor development and metastasis. Tumors that arise in response to overexpression of IRS-1 and IRS-2 have increased β-catenin signaling as evidenced by the upregulation of downstream target genes cyclin D1 and c-Myc [76]. These *in vivo *studies confirm the oncogenic potential of both of these adaptor proteins.

IRS-1 has been implicated in the development of medulloblastomas through an interaction with the T-antigen of human polyomavirus JC (JCV T-antigen). Medulloblastoma cell lines and biopsies express high levels of the IGF-1R and IRS-1, the latter of which co-localizes with the JCV T-antigen in the nucleus [164]. Disruption of the interaction between IRS-1 and the JCV T-antigen using a dominant negative mutant of IRS-1 inhibits the anchorage-independent growth and survival of JCV T-antigen transformed medulloblastoma cells [164]. More recently IRS-1 and IRS-4 have been shown to play a role in transformation by adenovirus 5 early region 1A (Ad5E1A) by binding to the Ad5E1A protein [165]. Ad5E1A association with the IRS proteins results in increased IRS tyrosine phosphorylation and subsequent constitutive activation of the PI3K/Akt signaling pathway.

### Nuclear functions for the IRS proteins

The majority of studies that have investigated IRS function in cancer have focused on their role as cytoplasmic adaptor proteins. However, there is accumulating evidence that the IRS proteins may also have important functions in the nucleus. As mentioned above when discussing the transforming potential of the IRS proteins, IRS-1 co-localizes with the SV40 and JCV T-antigens in the nuclei of transformed cells [166,167]. Independently of any oncogenic stimulus, IGF-1 stimulation can also promote the nuclear localization of IRS-1 [168]. Recently, a positive correlation between IRS-1 nuclear expression and a more well-differentiated, non-metastatic phenotype for ductal breast cancer was reported [46]. These findings provide additional evidence that the IRS proteins may have distinct functions that are dependent upon their localization within the cell and that the activity of these adaptor proteins can be regulated by recruitment to or exclusion from a specific intracellular compartment.

With regard to function in the nucleus, IRS-1 can be detected on promoter sequences of several genes, including c-myc, Cyclin D1 and ER target genes [169,170]. Studies in breast carcinoma cells reveal interactions between IRS-1 and the transcription factors β-catenin, ER-α and the androgen receptor (AR) [76,169,171,172]. Interactions of IRS-1 with β-catenin and AR positively regulate transcription, whereas IRS-1 antagonizes ER-dependent expression of genes that contain estrogen response elements (EREs) [169]. Although IRS-1 is capable of directing nuclear localization of β-catenin, ER-α is responsible for the nuclear translocation of IRS-1 in response to estrogen treatment [169]. IRS-1 also interacts with upstream binding factor-1 (UBF1) and regulates RNA polymerase activity to increase ribosomal RNA synthesis [173].

A role for IRS-1 in DNA repair has also been reported. In normal cells, IRS-1 binds to Rad51, a key enzyme in homologous recombination-directed DNA repair (HRR), and regulates its recruitment into the nucleus in response to agents that cause double strand breaks [174]. Phosphorylation of IRS-1 on tyrosine residues disrupts its interaction with Rad-51 and allows Rad51 to translocate into the nucleus to initiate DNA repair. In the absence of IGF-1 signaling, the IRS-1/Rad51 interaction is maintained and repair is impeded [174]. In medulloblastomas, IRS-1 translocates to the nucleus with ERβ or the JCV T-antigen, where it interacts with Rad51 and prevents HRR, rendering these tumors more sensitive to genotoxic agents such as cisplatin [175,176].

## Conclusion

The IRS proteins have been implicated in contributing to all stages of cancer, from initiating events to metastatic progression. However, there is still much to be learned about the mechanisms by which each of the IRS proteins differentially contribute to tumor cell function and the manner in which their expression and function are regulated. Understanding how the tumor microenvironment and other oncogenic signaling pathways impinge upon the IRS proteins to influence their signaling functions is essential for the future development of these adaptor proteins as either predictive markers for drug responsiveness or as therapeutic targets themselves. Given that IRS-1 and IRS-2 mediate distinct cellular responses to IGF-1stimulation, their relative expression levels and functional status are likely to impact the response of tumors to therapies that target the IGF-1 signaling axis. Assays that can determine not only the expression of the IRS family members but also their functional status will need to be developed to identify patients that are likely to be responsive to this targeted therapy and what outcomes should be anticipated.

The contribution of the IRS proteins to drug resistance is another important area for future investigation. The expression and function of the IRS proteins are tightly regulated by negative feedback loops, many of which are disrupted by drugs that target oncogenic signaling pathways. For example, prolonged inhibition of EGFR or MAPK signaling prevents the MAPK-mediated degradation of IRS-1, which increases IGF-1R signaling and resistance to EGFR-inhibition therapy [177,178]. Likewise, resistance to rapamycin treatment can occur through the upregulation of IRS-1-mediated PI3K signaling that occurs due to the disruption of S6-kinase-mediated degradation of the IRS proteins [138,143]. Taken together, these studies reveal the importance of negative feedback regulation of the IRS proteins and underscore the importance of assessing IRS expression and function when designing new therapies that will disrupt these feedback mechanisms.

## Competing interests

The authors declare that they have no competing interests.

## Authors' contributions

All of the authors contributed to the writing of this manuscript. All of the authors have read and approved the final manuscript.
